# Editorial: Evaluating supervision and research leadership in promoting responsible research

**DOI:** 10.3389/frma.2025.1670586

**Published:** 2025-09-04

**Authors:** Tamarinde Haven, Dena Plemmons, De-Ming Chau

**Affiliations:** ^1^Department of Methodology and Statistics, Tilburg University, Tilburg, Netherlands; ^2^Graduate Division, University of California, Riverside, Riverside, CA, United States; ^3^Department of Biomedical Sciences, Universiti Putra Malaysia, Serdang, Malaysia

**Keywords:** research integrity, mentoring, supervision, leadership, responsible conduct of research, open science, research ethics

This editorial provides an overview of the contributions to the Research Topic, placing them within the broader research context. It also reflects critically on their implications, limitations, and potential directions for future research. The four contributions in this Research Topic are recent advances in measuring, conceptualizing, and implementing responsible supervision and leadership within research environments to foster responsible research. They range from psychometric assessment to conceptual frameworks, practical roadmaps, and normative reflections—clarifying what responsible leadership looks like, how to evaluate it, and what enables it to flourish.

## 1 Grounding evaluation in empirical measurement: CARES Climate Survey

Martinson et al.'s development of the CARES Climate Survey fills a gap by offering a psychometrically validated 22-item scale regarding the interpersonal climate. It captures different, but connected key aspects of the interpersonal climate: civility, interpersonal accountability, conflict resolution, and harassment-responsiveness in research work-units. Built from two large samples (*N* = 1,384; *N* = 868) of researchers across U.S. contexts, CARES addresses the need to assess whether supervisors and lab leaders create respectful and safe interpersonal climates, highlighting interpersonal dimensions often overlooked in traditional research integrity assessments. CARES items tap into the interpersonal dimensions both at the level of interaction between colleagues, as well as influence from lab leaders, and the broader institutional context. Its outcomes could inform behavior change interventions, be used as an evaluation of the research culture, and help highlight high-performing labs that could serve as role-models.

## 2 Conceptual framework: Leadership, Management, Mentoring (LMM)

Mcintosh and Antes conceptualize supervision and research leadership through their proposed Leadership, Management, and Mentoring (LMM) framework, delineating three interwoven roles of research leadership: culture-building (*leader*), ensuring daily operational rigor (*manager*), and fostering individual growth (*mentor*). Effective LMM within a research organization enhances collaboration, communication, trust and openness, which leads to improved research integrity such as data integrity and reproducible science. The authors proposed several models and measurement tools for effective LMM evaluation. One of these methods is a bottom-up approach that relies on peer review, in which research leaders assess LMM performances via the lens of other researchers. While various assessment methods have advantages and disadvantages, the institutional leadership is responsible for applying these tools carefully based on the context of their respective research institution.

## 3 Training in practice: A European roadmap

Lacey et al. offer a roadmap for training supervisors and leaders at the European level, identifying institutional practices, curricula, and organizational support mechanisms. Using a creative virtual marketplace approach, their study provides empirical insights into which training features—peer networks, scenario-based workshops, reflective exercises—prepare supervisors to foster responsible research. This research found that supervision training should be mandatory and designed according to the needs of different disciplines. In addition, the issue of power imbalance between junior and senior research team members is an area that needs to be addressed if the research team were to encourage a culture of responsible research. Crucially, they emphasize embedding training into institutional promotion criteria, thereby aligning supervision incentives with responsible research outcomes.

## 4 Perspective: Role modeling and empowerment

Loon et al. offer reflections on supervision as role modeling, advocating for an empowerment perspective, such as articulated in the work of Freire (1970), which supports the development of both supervisors and supervisees to become critically aware of power imbalances which can compromise the research environment/research practice; to feel safe and responsible to address integrity issues proactively; and to engage in communication and reflection in an on-going process of dialogical learning. The authors identify what they find to be key aspects of role modeling in supervision, and discuss how an empowerment perspective, and training programs based on the same, can support the development of necessary capacities in each of these aspects, and work together toward a more positive, inclusive, and responsible research culture.

## 5 Gaps and suggested use cases

We do want to acknowledge that the contributions in this Research Topic are by authors from the Global North, and represent work based on engagement with stakeholders or participants primarily in that same setting. This underscores the limits in generalizability of results, and transferability of approaches; measurement of good supervision—indeed, what *defines* good supervision—may well look different in the Global South. It should be noted that the CARES instrument was validated using a diverse sample, with close to one third of their second sample identified as non-white. We encourage more and further cross-cultural work in fair collaboration with researchers from the Global South on supervision and mentoring in the research context, and measurement of the same, in this space.

We also acknowledge the lack of empirical evidence for the successful implementation of these supervision or leadership assessment tools. In other words, there is currently limited empirical evidence regarding whether these tools enhance responsible research, underscoring a valuable opportunity for future investigation. One potential methodological approach to investigate effectiveness regards “natural experiments” (Sieweke and Santoni, 2019). In this design, researchers use an exogenous event, e.g., an implementation of a new leadership policy at a particular institution, as if it was a condition randomly assigned to otherwise comparable groups (where the comparator would be a similar institution where no new leadership policy was implemented).

Finally, what we find in the research articles in this topical Research Topic, and in much of the research into responsible conduct of research generally, is a focus on the individual researcher (as researcher and/or as supervisor/mentor/leader) and the results of those measurements are confined to individual researchers or team leaders. Of course, an ethical research culture is not sustainable if practiced or created by, or connected to, one individual. We are curious about research which focuses on *institutional* commitments to the responsible conduct of research, and how evaluations might effectively assess whether institutional leadership is committed to an ethical research culture. This might involve some form of integrative assessment, where measures on different levels (the individual, the research group, the faculty, the institution) are combined. The CARES instrument is one tool that leadership can use to see, for instance, how committed researchers are to creating a responsible research culture in their own labs/research groups (Martinson et al.), but it is not specifically targeting deans, faculty heads, or even rectors to reflect on any institutional practices which might foster or obstruct an ethical research culture at the level of the institution.

## 6 Concluding remarks

We hope these contributions, as well as the recognition of the limitations of work in this area, serve as a catalyst for collaborative development across contexts and cultures with a goal of promoting broad and systemic change in how we support responsible research through responsible supervision and leadership. By modeling responsible supervision and leadership, we can equip future generations of researchers to navigate the research landscape and to conduct responsible research.
